# A feasibility randomised controlled trial of Novel Activity Management in severe ASthma-Tailored Exercise (NAMASTE): yoga and mindfulness

**DOI:** 10.1186/s12890-021-01436-3

**Published:** 2021-02-27

**Authors:** Sarah A. Hiles, Paola D. Urroz, Peter G. Gibson, Adam Bogdanovs, Vanessa M. McDonald

**Affiliations:** 1 Centre of Excellence in Severe Asthma and Priority Research Centre for Healthy Lungs, University of Newcastle, University Drive, Callaghan, NSW 2308 Australia; 2 Department of Respiratory and Sleep Medicine, John Hunter Hospital, New Lambton Heights, NSW Australia; 3 Yoga For All, Newcastle, NSW Australia

**Keywords:** Severe asthma, Yoga, Mindfulness, Asthma management, Health-related quality of life, Exercise, Physical activity, Sedentary

## Abstract

**Background:**

Physical inactivity is common in severe asthma and associated with poor health outcomes. New approaches are needed to address physical inactivity in this group.

**Objective:**

To examine whether yoga and mindfulness improves health-related quality of life (HRQoL) compared with a minimal active control group and collect feasibility data to inform future studies.

**Methods:**

Over 12-weeks, adults with severe asthma were recruited. Participants were randomised 2:1 to parallel yoga or control groups. All participants received an activity tracker. The yoga group received tailored group classes twice a week for 16-weeks with a qualified yoga instructor. The control group set activity goals with a research officer and received eight progress calls. Outcomes were assessed at 16-weeks. Primary outcome was St George’s Respiratory Questionnaire (SGRQ). Secondary outcomes included asthma control, physical activity, breathlessness, and inflammation. Face-to-face qualitative interviews were conducted to determine acceptability.

**Results:**

There were 15 participants randomised to yoga (mean 67 years; 60% female) and 9 to control (68 years; 56% female). Planned comparisons indicated the yoga group had greater SGRQ improvement than the control group. There was little change in secondary outcomes. Moderate-vigorous activity increased substantially in the control group. Participants found the intervention acceptable; key barriers and facilitators were social connection, the setting, addressing breathing and asthma symptoms, changing their mindset, and the intersection of different elements.

**Conclusion:**

A yoga and mindfulness intervention was feasible, acceptable to patients and improved HRQoL. The findings will inform design of much needed future research into physical activity interventions for severe asthma.

*World Health Organization International Clinical Trials Registry Platform* The study was registered under the Australian New Zealand Clinical Trials Registry (ANZCTR) on the 26th of November 2018, Trial ID ACTRN12618001914257.

**Supplementary Information:**

The online version contains supplementary material available at 10.1186/s12890-021-01436-3.

## Introduction

People with severe asthma have a range of comorbidities and risk factors that worsen prognosis and impair quality of life [1]. Physical inactivity and sedentary behaviour are common and largely ignored, with few studies characterising these behaviours and fewer interventions targeting activity in severe asthma [2, 3]. Physical inactivity is associated with negative health outcomes, including obesity, anxiety and depression, which are common comorbidities in severe asthma [1, 3, 4]. To date there have been no targeted interventions for people with severe asthma to simultaneously address these multiple risk-factors.

Given the benefits of physical activity in healthy populations [5, 6], asthma [7, 8] and COPD [9, 10], it is likely that increasing physical activity and reducing sedentary time will lead to improved outcomes for people with severe asthma. However, this can be complex in severe asthma as exercise can increase symptoms and lead to fear of exercising and increased sedentary behaviour [10]. Yoga is a mind–body practice that involves breathing, postures and stretches, and meditation. These practices have demonstrated health benefits, particularly for cardiometabolic and mental health [11, 12]. Although high quality studies are lacking, evidence suggests that yoga and mindfulness have positive effects on asthma-related quality of life when compared with usual care in mild to moderate disease, with mixed or limited evidence as to whether yoga influences spirometry, biomarkers or asthma control [13–15]. Typically, people with severe asthma have been excluded from these studies, yet this population have the greatest deficits in quality of life and most to gain.

The primary aim of this study was to investigate whether tailored yoga and mindfulness classes improve health-related quality of life of people with severe asthma, compared with a minimal active control group focused on physical activity goal-setting. We also aimed to investigate the intervention’s feasibility and acceptability from the patients’ perspective, and its effect on other important clinical outcomes, including physical activity, breathlessness, symptoms of anxiety and depression, sleep quality and systemic inflammation. We hypothesised that yoga and mindfulness classes will lead to significant improvements in health-related quality of life and other important clinical outcomes.

## Methods

### Design

This was a single-centre parallel-group pilot randomised controlled trial (RCT) with 2:1 allocation to the yoga intervention group or minimal active control group. The 2:1 allocation was to ensure there were enough participants to run two classes effectively and gain sufficient qualitative data regarding the intervention’s feasibility and acceptability. Concealed random allocation was employed. A statistician independent of the study team generated the random sequence using an online generator [16] (strata: male, female; block size: 3, 6) and randomly allocated participants to groups once the participant was confirmed eligible by the principal investigator following their baseline assessment. Primary and secondary outcomes were assessed before and immediately post-intervention. Participant-blinded and investigator-blinded behavioural intervention was not possible and self-report outcome measures cannot be considered blind.

The study was designed according to SPIRIT guidelines and was conducted in accordance with the principles of Good Clinical Practice. Ethical approval was obtained (HNEHREC 2018/ETH00338) and all participants provided written informed consent. The trial was registered on the Australia New Zealand Clinical Trials Register (ACTRN12618001914257).

### Setting

Study assessments and the yoga intervention were conducted at the Hunter Medical Research Institute, Australia.

### Participants

During a 12-week period (16/11/2018–8/2/2019), participants were recruited via the John Hunter Hospital Department of Respiratory and Sleep Medicine’s ambulatory care clinics and research database, and via general/social media advertisement. We included adults (≥ 18 years) with evidence of variable airflow limitation in the last 10 years (bronchodilator response ≥ 12% or airway hyper-responsiveness or peak flow diary (variation ≥ 15% or > 50 ml), and severe asthma according to the European Respiratory Society (ERS)/American Thoracic Society (ATS) taskforce definition [17]: asthma requiring high-dose inhaled corticosteroids (> 1000 μg beclomethasone equivalent [18]) with a second controller to prevent uncontrolled disease or disease that remains uncontrolled despite therapy. Detailed inclusion and exclusion are in Additional file 1: Supplementary methods and additional results.

### Interventions

#### Yoga and mindfulness group

Detailed information is available in Additional file 1: Supplementary methods and additional results. Briefly, the intervention group participated in two supervised 75-min group classes of yoga and mindfulness per week for 16-weeks in a private room during office hours (up to 8 participants/class), in addition to usual care. An accredited yoga practitioner designed and delivered the programme, in collaboration with the investigator team. Classes focused on increasing movement, controlling breath, and meditation to improve mindfulness, and were designed to be suitable regardless of experience, ability and physical limitations. The instructor explained how to perform the exercises, described precautions and encouraged participants to apply the lessons at home. An experienced clinical researcher was present before and after sessions to monitor participant’s ability to participate in the class and was on-call in case of adverse events. Participants were advised to self-administer salbutamol before sessions to prevent exercise-induced bronchospasm.

At their first class participants received an activity tracker (Fitbit Charge 2 [Fitbit, Inc], or pedometer for participants without smartphones) and were encouraged to progressively increase their daily steps according to an algorithm [19], up to 10,000 steps/day. Participants were also given information sheets (physical activity guidelines [20], SMART goal-setting [21], exercise and severe asthma [22] and mindfulness [23]), a printed calendar to track their activity goals, and a BORG Dyspnoea Scale [24] to monitor dyspnoea during physical activity.

#### Control group

The control group received a minimal physical activity goal-setting intervention supported through telephone contact with an exercise physiologist research officer, in addition to usual care. Participants were mailed their activity tracker, the same printed documents as the yoga group, and a workbook to record goals and progress. During the first call (average 14:27 min), participants set two SMART goals: one regarding daily steps according to an algorithm [19] and another of their choosing regarding physical activity or wellbeing. The research officer called the participant every 2 weeks for 16-weeks (average 7:17 min/call) to check on progress toward goals, provide encouragement, and adjust goals if necessary. More information is available in Additional file 1: Supplementary methods and additional results.

### Outcome assessment

#### Assessments

Participants underwent face-to-face clinical assessments before randomisation and immediately after the intervention period (16-weeks) to assess primary and secondary outcomes. Additionally, demographic information, asthma duration, smoking history, respiratory and other medication use, and vital signs were assessed at baseline. Participants were telephoned every 3 months up to 9 months post-intervention to assess exacerbations since their last assessment.

#### Primary outcome

The pre-specified primary outcome was St George’s Respiratory Questionnaire (SGRQ) [25], a valid self-reported health-related quality of life measure in severe asthma [26]. SGRQ describes health-related quality of life globally and in domains *symptoms*, *activity* and *impact*. The minimal clinically important difference is a minus 4-unit change [27].

#### Secondary outcomes

Symptom control was assessed via the Asthma Control Questionnaire-5 (ACQ-5) [28]. Past-year moderate and severe exacerbations as described by ERS/ATS guidelines [17] were self-reported. Peripheral blood was collected and analysed for full blood count. Blood serum was analysed for high-sensitivity C-reactive protein (Pathology North, Australia). Fractional exhaled nitric oxide (FeNO) was measured (NIOX VERO). Pre- and post-bronchodilator spirometry (Medigraphics) was completed (Global Lung Initiative predicted values [29]). Body weight and height were measured to calculate body mass index. Percentage body fat and percentage lean muscle mass were analysed by bioimpedance scales (InBody 720). Physical activity intensity and duration, steps per day, and sedentary time were assessed using ActiGraph wGT3X-BT triaxial accelerometers (ActiGraph LLC, Pensacola, FL). Participants wore the accelerometer on their waist 24 h/day for 8 consecutive days, and data were processed as previously described [3]. Participants completed the 6-min walk test [30] Participants completed self-report measures:

- Pittsburgh Sleep Quality Index [31];
- International Physical Activity Questionnaire-Short Form [32];
- Hospital Anxiety and Depression Scale [33];
- Dyspnoea-12 [34];
- EQ-5D [35] to assess health status;
- Cognitive and Affective Mindfulness Scale-Revised [36]; and
- Friendship Scale [37] to assess social isolation.

Charlson Comorbidity Index [38] was calculated from self-reported medical history.

#### Adherence

Attendance was recorded before each yoga session. Control group phone calls were also logged.

#### Semi-structured interviews

We invited all participants to a face-to-face semi-structured interview. The interview occurred within 2 months of the intervention with an author who did not deliver the intervention (SAH). Participants were asked open-ended questions from an interview guide about their physical activity, perceived benefits and weaknesses of the intervention, and adherence to the intervention. Interviews were audio-recorded and transcribed verbatim.

### Sample size

This pilot trial was designed test the study protocol, determine acceptability, and derive outcome effect size data that will inform the sample size calculation for a larger trial [39]. Therefore, no formal power calculation is required [39]. Nevertheless, our initial recruitment target was 54 participants, based on detecting a 6-unit difference in the change in SGRQ between treatment and control groups (SD = 12; within-subject r = 0.8, α = 0.05, β = 80%). Due to a short 3-month recruitment window that coincided with the Christmas-New Year period, 25 participants were assessed for entry to the study.

### Data analysis

Quantitative analyses were conducted in Stata IC/15 (StataCorp LLC). Data were analysed on intention-to-treat basis, including all available data in the analysis. Tests were two-sided with *p* < 0.05 considered statistically significant. Descriptive statistics were calculated for demographic and clinical characteristics. Correlation between attendance and SGRQ was calculated for the intervention group. For primary outcome analysis, a restricted maximum likelihood (REML) linear mixed model was conducted, with outcome of SGRQ at baseline and post-treatment assessment, and predictors of treatment condition (intervention vs control), time (baseline vs post-treatment) and their interaction. Given the small sample size and low power to detect interaction effects, planned comparisons examining SGRQ change for each group were conducted. Secondary outcomes were assessed using REML linear mixed models or negative binomial regression for exacerbations. Given this is a pilot trial, analyses focus on confidence interval estimation [39].

Transcribed interview data underwent a manifest qualitative analysis, facilitated with NVivo 12 (QSR International Pty Ltd). Deductive coding was completed, based on the interview schedule. Inductive coding was undertaken concurrently to ensure all content was coded. Codes and themes were discussed by two authors (SAH, VMcD). Yoga group interviews were analysed for the current study.

## Results

### Recruitment

**Fig. 1:**
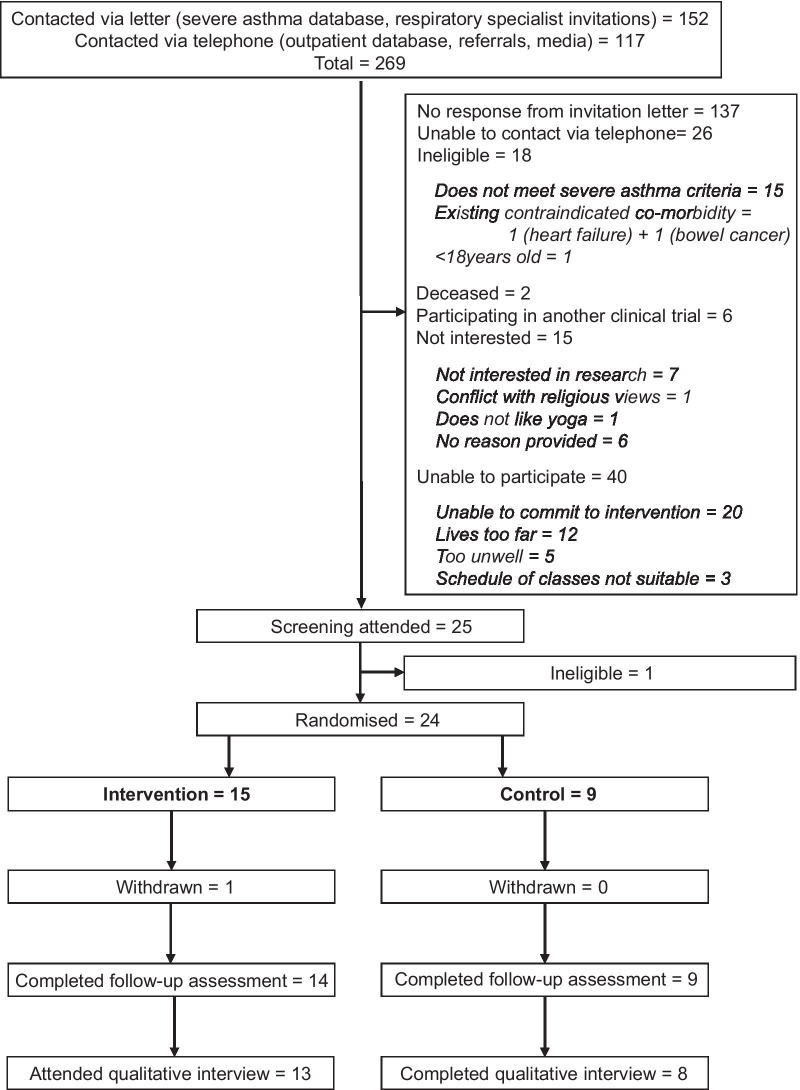
Flow diagram indicting the number of participants contacted, screened, eligible and completing the study

During the 12-week recruitment period, 269 potential participants were contacted via telephone or invitation letter (Fig. 1). From this, 25 individuals were assessed: one was ineligible and 24 were randomised. Twenty three participants (96%) completed the follow-up outcome assessments and 21 (88%) completed the qualitative interview. The most common reasons for not attending the screening visit were non-response to the invitation letter (N = 137) or being uncontactable via telephone (N = 26). Only 15 reported they were not interested in participating. Among individuals who were interested but said they were unable to participate, reasons were inability to commit to the intervention (N = 20), lived too far away (N = 12), were too unwell (N = 5) or found the schedule of classes unsuitable (N = 3).

### Characteristics of participants

Baseline demographic and clinical characteristics were largely balanced between the intervention and control groups (Table 1), although the control group had later diagnosis of asthma, higher FeNO, greater steps per day, greater self-reported physical activity, greater walk distance and lower anxiety (Table 2). Participants were on average 67 years (SD = 9) and more likely to be female (58%) than male. Baseline health-related quality of life was impaired (SGRQ 42 ± 18). Participants completed a median of 4387 (interquartile range 3524, 6732) steps and 14 (8, 34) minutes of moderate-vigorous activity per day, which is below Government recommendations [20].

**Table 1:** Baseline characteristics

	Yoga group	Control group	*p*
N	15	9	
Current age in years, mean (SD)	67 (9)	68 (8)	0.86
Female, N (%)	9 (60%)	5 (56%)	0.83
Employed full- or part-time, N (%)	5 (33%)	3 (33%)	1.00
Ex-smoker, N (%)	5 (33%)	3 (33%)	1.00
Age of asthma diagnosis, median (IQR)	11 (3, 48)	40 (22, 45)	0.20
Number of exacerbations in the last 12 months, median (IQR)	2 (1, 4)	3 (2, 3)	0.21
CCI, median (IQR)	0 (0, 4)	0 (0, 2)	0.59
Current medication use			
ICS/LABA, N (%)	15 (100%)	9 (100%)	N/A
ICS Beclomethasone equivalent dose, median (IQR)	2000 (1000, 2000)	2000 (1000, 2000)	0.36
Biological therapies, N (%)	10 (67%)	7 (78%)	0.56
LAMA, N (%)	8 (53%)	5 (56%)	0.92
LTRA, N (%)	0 (0%)	1 (11%)	0.19
Maintenance oral corticosteroid, N (%)	5 (33%)	2 (22%)	0.56

*CCI* Charlson Comorbidity Index, *ICS* inhaled corticosteroid, *IQR* interquartile range, *LABA* long-acting beta-agonist, *LAMA* long-acting muscarinic antagonists, *LTRA* leukotriene receptor antagonist, *N* number, *N/A* not applicable

**Table 2:** Group means or medians for secondary outcomes pre- and post-intervention, and mean within-person change in secondary outcomes, with 95% confidence intervals (CI)

	Yoga group			Control group		
	Pre, mean/median (CI)	Post, mean/median (CI)	Within-person change, mean (CI)	Pre, mean/median (CI)	Post, mean/median (CI)	Within-person change, mean (CI)
Asthma control and quality of life						
ACQ5, median (CI)	1.4 (0.6, 2.0)	0.5 (0.2, 1.8)	− 0.4 (− 1.0, 0.1)	0.8 (0.0, 2.1)	0.6 (0.2, 2.2)	0.2 (− 0.1, 0.5)
SGRQ—Total score, mean (CI)	44 (32, 55)	35 (21, 48)	− 10 (− 21, 1)	40 (29, 51)	38 (26, 49)	− 3 (− 11, 6)
SGRQ—Activity, mean (CI)	56 (42, 70)	46 (30, 61)	− 12 (− 25, 2)	56 (32, 79)	49 (26, 72)	− 7 (− 18, 5)
SGRQ—Symptoms, mean (CI)	48 (31, 65)	41 (23, 59)	− 8 (− 22, 6)	40 (25, 55)	46 (32, 61)	6 (− 2, 15)
SGRQ—Impacts, mean (CI)	35 (24, 47)	27 (14, 40)	− 9 (− 19, 0)	31 (21, 42)	28 (17, 40)	− 3 (− 16, 10)
EQ-5D—Your health today, median (CI)	80 (62, 88)	80 (61, 95)	7 (− 1, 15)	85 (67, 93)	90 (70, 95)	1 (− 7, 10)
Number of exacerbations in the 12 months before baseline assessment (“*pre*”) or the duration since baseline assessment [median 56 weeks] (“*post*”), median (CI)	2 (1, 3)	1 (1, 2)	0 (− 2, 2)	3 (2, 3)	2 (1, 3)	− 1 (− 3,1)
Physical activity						
Actigraph: Steps per day, mean (CI)	4883 (3749, 6017)	3923 (2828, 5018)	− 101 (− 2073, 1871)	5652 (3367, 7937)	6599 (3963, 9234)	682 (− 912, 2277)
Actigraph: Sedentary minutes per day, mean (CI)	791 (656, 925)	828 (669, 986)	− 100 (− 265, 65)	792 (586, 999)	762 (569, 955)	12 (− 113, 136)
Actigraph: Moderate-vigorous physical activity mean min/day, median (CI)	14 (8, 31)	17 (8, 23)	2 (− 14, 18)	14 (5, 64)	45 (15, 71)	11 (0, 21)
Actigraph: Light physical activity mean min/day, median (CI)	161 (137, 175)	153 (102, 193)	12 (− 45, 69)	140 (124, 163)	142 (83, 168)	− 12 (− 46, 23)
IPAQ—Metabolic minutes per week, median (CI)	960 (198, 1600)	1302 (362, 2935)	752 (81, 1423)	2048 (105, 4073)	2772 (747, 4916)	1108 (− 1244, 3461)
Post-bronchodilator lung function						
% predicted FEV1, mean (CI)	75 (61, 88)	76 (64, 88)	3 (− 3, 10)	72 (56, 87)	73 (51, 96)	0 (− 6, 6)
% predicted FVC, mean (CI)	91 (76, 105)	92 (78, 106)	3 (− 5, 11)	90 (81, 98)	88 (74, 101)	− 1 (− 10, 8)
FER, mean (CI)	0.64 (0.57, 0.72)	0.65 (0.57, 0.74)	0.01 (− 0.02, 0.04)	0.62 (0.48, 0.76)	0.64 (0.48, 0.80)	0.00 (− 0.03, 0.03)
Other physiological outcomes						
Blood eosinophils, median (CI)	0.1 (0.0, 0.2)	0.1 (0.0, 0.3)	0.0 (− 0.1, 0.0)	0.1 (0.0, 0.2)	0.1 (0.0, 0.2)	− 0.1 (− 0.2, 0.1)
FeNO, median (CI)	20 (10, 46)	26 (18, 45)	1 (− 5, 8)	43 (21, 91)	36 (13, 69)	− 6 (− 18, 6)
hs-CRP, median (CI)	3 (1, 4)	3 (2, 5)	1 (− 1, 3)	1 (1, 10)	4 (1, 9)	− 6 (− 26, 13)
BMI, mean (CI)	32 (28, 35)	32 (29, 35)	0 (− 1, 1)	29 (24, 34)	29 (24, 35)	0 (0, 1)
BIA % body fat mass, mean (CI)	41 (35, 47)	42 (37, 48)	1 (0, 2)	37 (26, 47)	38 (28, 47)	1 (0, 2)
Total walk distance, mean (CI)	436 (384, 488)	453 (392, 513)	33 (− 2, 67)	526 (424, 628)	546 (441, 652)	20 (− 40, 80)
Other patient reported outcomes						
Dyspnea-12—total score (high score is greater symptoms), mean (CI)	11 (6, 15)	9 (3, 15)	− 4 (− 8, 1)	7 (2, 13)	10 (4, 17)	4 (− 2, 9)
HADS Anxiety score (high score is greater symptoms), median (CI)	6 (4, 9)	6 (2, 10)	− 1 (− 3, 1)	4 (0, 7)	4 (2, 7)	0 (− 2, 2)
HADS Depression score (high score is greater symptoms), median (CI)	3 (3, 8)	5 (1, 8)	− 1 (− 2, 0)	4 (1, 8)	1 (0, 6)	− 1 (− 2, 0)
Pittsburgh Sleep Quality Index total (high score is poorer sleep quality), mean (CI)	8 (6, 9)	8 (5, 10)	− 1 (− 3, 2)	7 (5, 9)	5 (3, 7)	− 1 (− 4, 1)
Friendship Assessment total score (high score is less social isolation), median (CI)	20 (18, 24)	20 (16, 22)	− 1 (− 4, 1)	23 (13, 24)	21 (17, 24)	− 1 (− 4, 2)
CAMS-R total score (high score is more mindfulness), median (CI)	33 (29, 35)	34 (28, 37)	2 (− 1, 5)	34 (21, 38)	37 (30, 40)	4 (− 2, 10)

*ACQ-5* Asthma Control Questionnaire-5, *BIA* bioimpedance analysis, *BMI* body mass index, *CAMS-R* Cognitive and Affective Mindfulness Scale – Revised, *CI* confidence interval, *FeNO* fractional exhaled nitric oxide, *FER* forced expiratory ratio, *FEV1* forced expiratory volume in 1 s, *FVC* forced vital capacity, *HADS* Hospital Anxiety and Depression Scale, *hs-CRP* high sensitivity C-reactive protein, *IPAQ* International Physical Activity Questionnaire, *SGRQ* St George’s Respiratory Questionnaire

### Attendance

Participants in the intervention group attended a median of 19.5/32 sessions (range 6–31). Attendance and change in SGRQ during the intervention were not associated (*r* = − 0.12, *p* = 0.694). Control group participants received a median of 9/9 telephone calls (range 7–9).

### Intervention efficacy

**Fig. 2:**
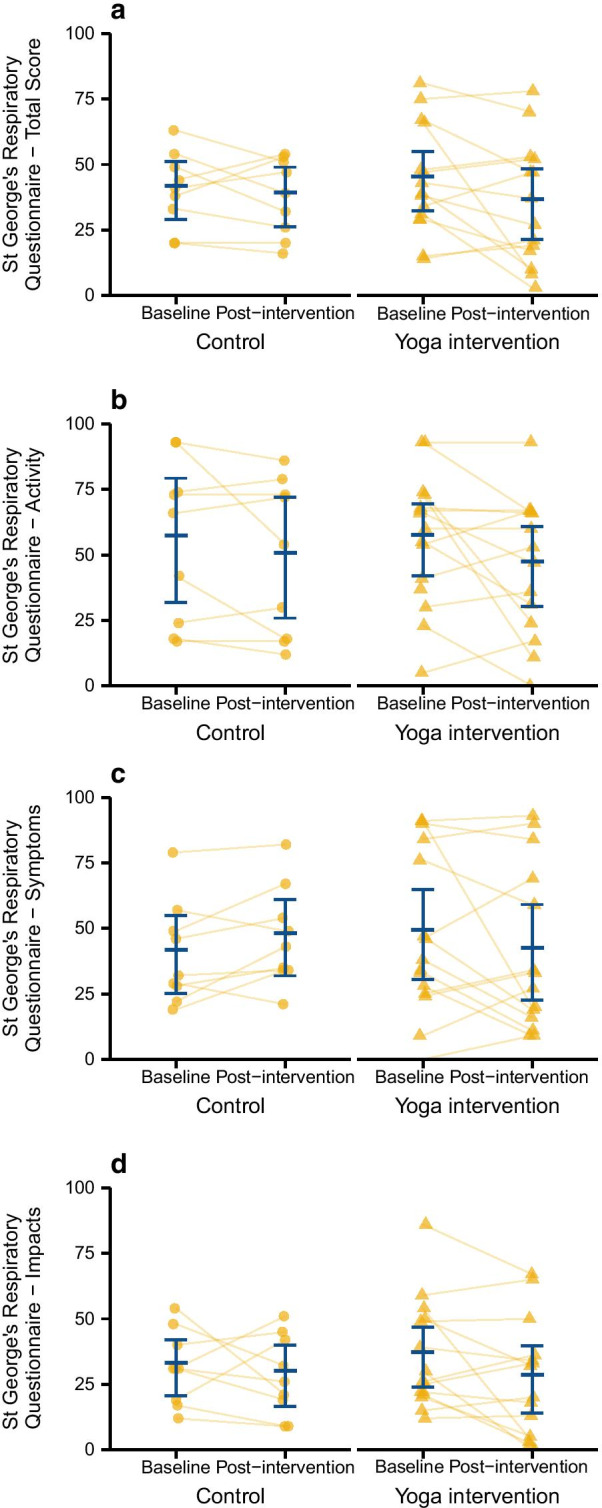
Primary outcome of St George’s Respiratory Questionnaire (SGRQ) in the yoga intervention and control groups before and after the intervention, for **a** total score; **b** activity domain, **c** symptom domain and **d** impact domain. Yellow circles or triangles are individual data values and blue bars indicate group mean or median and 95% confidence interval

For the primary outcome of SGRQ total score, there was no significant interaction between time (baseline vs follow-up) and group (control vs intervention) (*p* = 0.327). This was most likely due to low power. Planned comparisons of each treatment arm separately in the REML model indicated the yoga group had a greater decrease in SGRQ (marginal mean ± standard error − 9.4 ± 4.4, *p* = 0.032) than the control group (− 2.5 ± 5.5, *p* = 0.647; Fig. 2a). Examining SGRQ subscales, the greatest mean within-person change was in the Activity domain (Table 2, Fig. 2b–d).

**Fig. 3:**
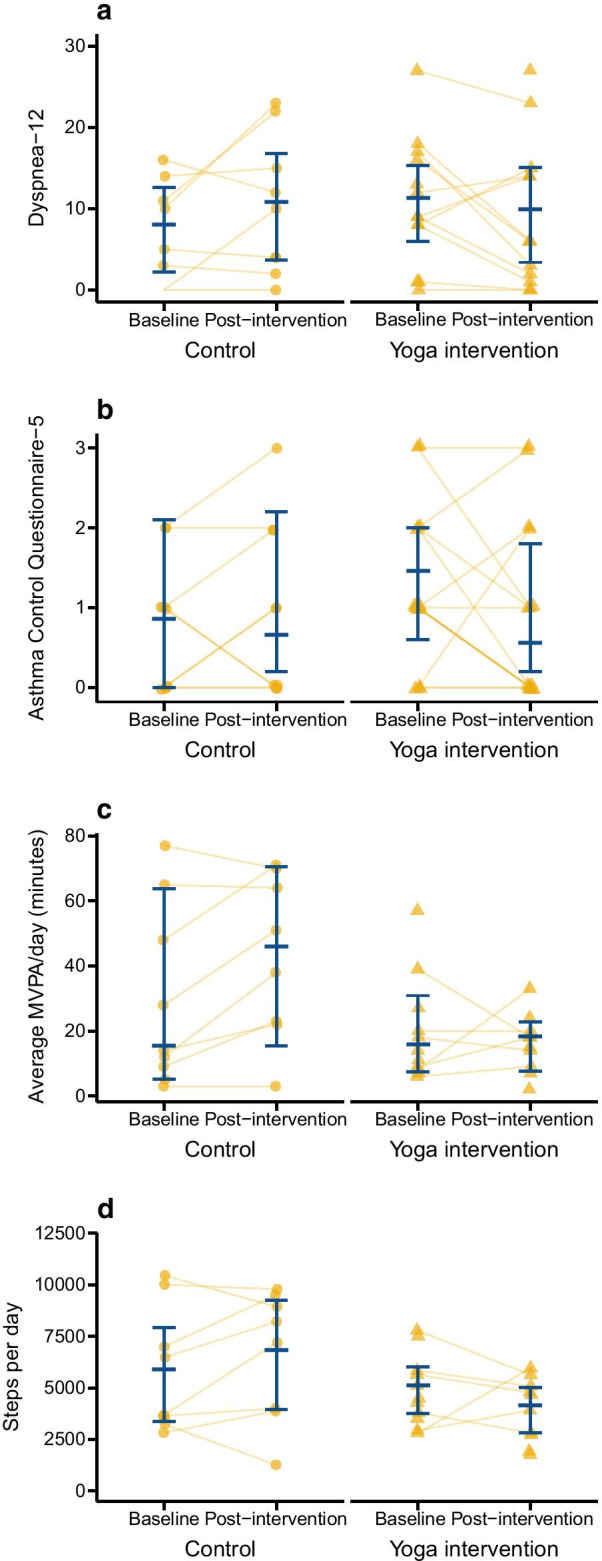
Secondary outcomes in the yoga intervention and control groups before and after the intervention for **a** Dyspnea-12 scores; **b** Asthma Control Questionnaire-5, **c** minutes of moderate-vigorous physical activity (MVPA) per day, and **d** steps per day. Yellow circles or triangles are individual data values and blue bars indicate group mean or median and 95% confidence interval

There was little change in most secondary outcomes (Table 2) and no statistically significant interactions between time and treatment group at *p* < 0.05, indicating change over time did not differ between groups. The control group slightly worsened in their Dyspnoea-12 scores, whereas the yoga group slightly improved (interaction *p* = 0.055; Fig. 3a). Examining the numeric changes in secondary outcomes, some of the most notable results included a reduction in median ACQ-5 in the yoga group compared with the control group (Fig. 3b); increase in daily moderate-to-vigorous activity in the control group compared with the yoga group (Fig. 3c); and increase in steps/day in the control group and similar magnitude decrease in the yoga group (Fig. 3d).

### Adverse events and safety

Adverse events in three individuals were suspected to be related to the interventions: shoulder impingement (control); vomiting/nausea/hypertension (yoga); and groin injury (yoga). The two yoga participants ceased the programme after their event. We recorded pre- and post-BORG dyspnoea ratings for 255 yoga sessions. Generally, there was either no change in ratings (N = 96) or a decrease after yoga (N = 141). Ratings increased on 18 occasions (7%), which was generally by 0.5 (N = 8) or 1 point (N = 7).

### Participant experience of the yoga intervention

**Fig. 4:**
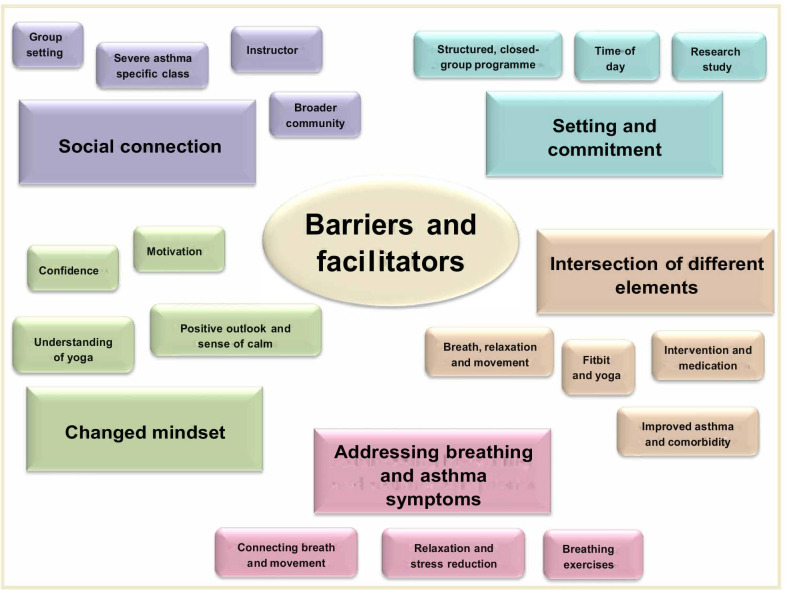
Summary of themes regarding barriers and facilitators of the intervention

Analysis of the qualitative interviews revealed five core themes regarding barriers and facilitators of the yoga intervention: social connection, setting/commitment, changed mindset, addressing breathing and asthma symptoms, and the intersection of different elements (Table 3; Fig. 4; briefly described here and detailed in Additional file 1: Supplementary methods and additional results). Social connection was highly regarded and largely facilitated the experience, specifically through the group-setting, asthma-specific group, connection with an instructor, and connection to the broader community. The setting provided barriers and facilitators pertaining to the structured closed-group programme, holding classes during office hours, and being involved in a research study. These factors increased commitment to attend and built skills yet led to disappointment when participants were unable to attend and may have curtailed ongoing practice beyond the trial. Participants described a changed mindset, which enhanced their experience. Specifically, they reported increased confidence and motivation to be active, an understanding of the value of yoga for asthma, and a more positive outlook. An important aspect for participants was that the programme addressed breathing and asthma symptoms. Most helpful elements were connecting breath with movement, learning relaxation skills for stress reduction, and the yogic breathing exercises reinforcing learnings from breath retraining in other settings. Finally, the intersection between different elements was a key facilitator in addressing multiple health and wellbeing concerns. Participants liked that the classes combined breath, relaxation and movement, and that the overall intervention combined a structured class with use of an activity tracker to motivate beyond class. For many participants, the intervention capitalised on recently improved health status, with the intervention encouraging them to push themselves. Participants reported various health improvements relevant to them; nevertheless, comorbidity sometimes led to negative, typically transient, effects during the yoga, which necessitated modifications to the exercises.

**Table 3:** Barriers and facilitators of the intervention from qualitative interviews with participants

Theme	Subtheme	Brief description	Illustrative quotes
Social connection	Group setting	The group setting assisted learning, was fun, and allowed participants to benchmark their abilities and progress against similar others	*F* age 72: “Because people can support each other and learn from each other and also I think it's supportive, you don’t feel as though—the focus is dispersed amongst the group, so you can hide in a way if you know what I mean.”
	Severe asthma specific class	Participants enjoyed speaking with others about their illness/medication experiences, felt less self-conscious about symptoms (eg, cough), and believed the shared experience of severe asthma enhanced togetherness and supportiveness	*F* age 78: “Well, it was very interesting doing the yoga with lots of other asthmatics. That was really good. Because a lot of people with asthma don’t really talk about it. So, you might know someone with asthma but you don't sit and yap about your asthma. Occasionally, you might. But things that came out in class about what this one or that one was doing, or how—it was good just to think you're not alone and that happens to someone else too.” *M* age 73: “Yeah, I didn’t worry about the cough. I did apologise a few times if I cough loud and that sort of thing, but I wasn’t the only one coughing… That’s why it felt more comfortable I think than just being in a group of people without those sort of problems.”
	Instructor	The instructor’s attitude, personality, attention, explanations and feedback enhanced learning and facilitated a positive class environment, increased retention and instilled a desire to do home practice. However, participants expressed hesitation at attending classes with a different instructor, which may be a barrier to continued practice	*F* age 49: “I've never done yoga before so I don't know what to expect from a yoga teacher, but he was just special, I mean seriously I fell in love with him, he was just wonderful. So patient and so good at communicating and taking it at our pace was the best thing, but also explaining the why, that was huge.” *F* age 78: “There's a few yoga places around, but I'd rather do—if [a new instructor] knows what [the intervention instructor] did, it would be better.”
	Broader community connection	Participants were able to talk about their experiences with yoga, or even practice yoga, with friends, family and others, in a way that may not have been possible with an exercise less well-known in the community	*M* age 75: “…I've quite openly been proud of the fact that I've been in yoga classes, talking to people that aren't involved in it, like my friends at the pub… So I've been comfortable to tell them that I've been a participant in it.”
Setting and commitment	Structured, closed-group programme	Participants appreciated that the programme gradually built on skills such that they could see changes in their ability and confidence. They felt a commitment to attend, or that it became a habit/routine to attend, which facilitated retention. However, participants described disappointment and frustration at being unable to attend due to illness or commitments, which was a barrier to continued practice	*F* age 61: “When we first started this I thought, 4 months, that’s a long time. Commitment wise from us but also from you guys to provide that, those classes for that amount of time. But what it did by having that sort of time, [the instructor is] sort of in your psyche now, in your head… I think that little voice is always going to be there now.” *F* age 70: “[I attended classes] for a month or something and then personal reasons, I couldn't do it…I was really devastated I couldn't come back for it, because I was really feeling I was getting a lot from it. So I don't know, maybe if I could have come longer, I would have gotten more, but I did feel benefit from what I did get.”
	Time of day	Participants suggested that working may be a barrier to attendance; participants found fitting the class around their schedules challenging	*F* age 61: “Well, I didn't like that I had to rush from [work] to get here. That was hard…So, if it was a little bit later it would have helped.”
	Research study	Participants prioritised attendance because it was a research study; they wanted to contribute to knowledge, trusted the people who put the programme together, and appreciated where it was conducted. Although this context increased retention, for some it was a barrier to ongoing practice as they viewed participating as a “one-time-thing”	*F* age 49: “…I thought I'm in a study, I need to maximise the effects of this study, I need to be full all in participation [sic]… you can't go into something like this half-heartedly and go oh well, you'll get your test results based on a half-hearted attempt. So, that's why I was so keen to do it and do everything that I'd been told to do.” *F* age 57: “Also the fact it was run via the hospital and it was science, there had to be some degree of thought I think behind that.”
Changed mindset	Confidence	Participants reported confidence to try exercises or activities of daily living that they usually would not attempt, or push themselves further in their activity than they usually would	*M* age 68: “I feel more confident to do various things and more confident that I'll be able to do them longer and more confident that…there'll be no unfavourable repercussions of pain subsequently.”
	Motivation	Participants reported motivation to exercise or do daily activities, often ascribed to awareness of their physical activity levels or the positive effects they experienced from physical activity	*M* age 73: “Yeah, well, besides all the extra walking, I’m finding that I feel more like I want to do things more so now, and the extra things I do, well, it’s probably what I was doing before, but this time with a bit more willingness to do it now.”
	Understanding of yoga	Many participants had pre-conceived ideas about yoga; the yoga exceeded their expectations and shifted attitudes—they developed an understanding of yoga and saw why it might be beneficial for asthma. All reported incorporating aspects of yoga in their daily routine	*F* age 57: “Yeah, I was I was dubious. I mean somebody of my age trying something totally new physically, it's like, whoa. And particularly when I generally have coffee [in a particular suburb near a yoga studio] and I do not fit the dynamic of the yoga class I see going into that room.” *F* age 61: “But this [yoga programme], knowing the purpose of it and actually being able to recognise how valuable it would be. Even though I had done yoga before, I didn’t really link it to be, [*participant’s name*] this could really help with how you manage your asthma.”
	Positive outlook and sense of calm	Many participants spoke about having a more positive outlook on life, decreased worry, and achieving a sense of “calm”	*F* age 70: “Mindset, yeah, opens your mind up to bigger and better things and, yeah, just be happy…It has made a really big difference in my attitude to daily life.” *F* age 62: “I think it was, yes, getting out of myself, coming to a place. The relaxation and just the working with my body to help it get all of the stress it had been under.”
Addressing breathing and asthma symptoms	Connecting breath and movement	Many found the focus on connecting breath with movement particularly helpful for managing day-to-day activity and intentional exercise. They thought that improved posture was helpful to broadening the chest and improving breathing	*F* age 62: “I went to the gym for all of those years and never thought about my breathing. If anything, when I was lifting heavy weights, I used to hold my breath. Now I've learnt to concentrate on my breath, while I’m doing those exercises, and I can lift heavier weights now, because I'm monitoring the oxygen going into my system.”
	Relaxation and stress reduction	Many found the focus on relaxation/stress reduction helpful for reducing anxiety and increasing a sense of control over symptoms of breathlessness, cough and wheeze	*F* age 49: “I find that the stressful side of things can bring on asthma, and this would teach people to destress.” *F* age 78: “Well, mainly with the yoga, I breathe better and I can manage my breathing so that if I get out of breath, I can sit down and concentrate and deep breathe and get out of the tight feeling and relax. Relaxation is very important.”
	Breathing exercises	Some connected the learning in the yoga class with previous experience of breathing exercises (eg, speech therapy), which they used as a tool to achieve more physical activity	*M* age 75: “I think I had most of the breathing exercises in practice before I went [to the yoga class], to be quite honest. It was only [the yoga instructor] giving me a reminder of what I'd been taught elsewhere to do.”
Intersection of different elements	Breath, relaxation and movement	Participants felt the combination of breath, relaxation and exercise/movement was beneficial	*F* age 70: “Having tried over the years quite a few [other types of exercise], this is the one I found that helped me the most in the body, the breathing, the mind, the attitude… It was a whole package… I found it gave me great confidence because I've never been one to have a lot of confidence, so it did give me confidence to try other things… not just sit at home and, as I said, get up and move and do things.”
	Fitbit and yoga	The Fitbit brought awareness of physical activity or motivated people to start their own new physical activity outside, and yoga was a way to learn different skills that facilitated physical activity outside of class	*F* age 49: “Having the combination of the yoga and the Fitbit to make me aware of my movement, that's a big thing to make me aware of every little bit counts, and you watch the little steps add up. So, that applies with when you're doing the yoga as well, you realise that every little bit adds up. Before Fitbit or yoga, I would avoid steps at all costs, I'm an elevator person. Hills, avoid hills, anything that was going to exert me because I knew that the result of exertion was asthma. In that regard, asthma was controlling me.”
	Intervention and medication	Participants used the intervention to get back to being physically active now that they felt better on new medication. Participants reflected that if they felt unwell, they were uncertain whether they could keep up with the classes or exercises	*F* age 61: “I think the medication definitely has made a big difference but this [intervention] on top of it reinforces the fact that it’s not a dead end, you know.” *M* age 68: “I might have struggled a bit with some of these yoga activities before the [monoclonal antibody therapy]… [the medication] freed me up to be active but these yoga activities are getting back my physical fitness.”
	Improved asthma and comorbidity	The programme had benefits for a range of comorbidities personal to each individual. People also reported their health, particularly pain, impeded their ability to follow the class. The tailored approach allowed them to overcome physical barriers; participants particularly appreciated the instructor being responsive to the needs of the class	*M* age 68: “Well, at the beginning of it I thought, I don't know how I'm going to go with this yoga, because it's hurting my back a bit; but I noticed at progressive times I came and I still did the activities where I didn't do them strenuously – I did them in a subtle, non-strenuous manner – and I found out that the recovery period got less and less to the point that now those activities don't hurt my lower back anymore.”

*F* female, *M* male

## Discussion

A 16-week yoga and mindfulness programme for people with severe asthma was feasible, acceptable and benefitted health-related quality of life. Although the small sample size limited the ability to detect statistically significant effects, participants in the yoga group significantly improved their health-related quality of life whereas participants in the active control group had no significant improvement. These data can inform larger studies of yoga interventions to assess whether numeric improvements in dyspnoea and asthma control are reliable. Larger studies of interventions involving activity tracking and goal-setting may also confirm whether physical activity improvements observed in the control group are reliable. When considering how to test and implement exercise interventions for people with severe asthma into the future, qualitative interviews highlighted the following potentially important components: forming social connections, an appropriate setting/structure, changing mindsets, addressing breathing and asthma symptoms, and combining multiple elements into one intervention package.

Participants reported impaired health-related quality of life at baseline, which improved for participants in the yoga group. The SGRQ Activity subscale shifted most. This subscale characterises activities that are caused or limited by breathlessness. The quantitative findings also suggested numeric (although small) improvements in dyspnoea and asthma control in the intervention group, relative to the control group. Previous studies in generally mild-to-moderate asthma similarly indicate that yoga improves health-related quality of life [13–15]. In studies of asthma and other diseases, yoga has also improved exercise capacity, physical limitation, fatigue, breathlessness, strength, mental health, and social connectedness [13–15, 40–44]. Consistent with the quantitative findings, participants described health and wellbeing benefits during the qualitative interviews, particularly in their breathing and ability/motivation to be active during daily life. Participants identified the most helpful components to improve breathing were learning to connect breath with movement, stress-reducing relaxation skills, and yogic breathing exercises. A change in mindset, namely increased confidence, motivation and understanding of how yoga may benefit their asthma, helped improve their perceived activity levels. However, quantitative physical activity values did not increase and steps/day decreased, possibly because low-intensity yoga displaced more active exercise or participants were prioritising relaxation activities. Participants who dropped out of the intervention due to injury or illness may have also contributed lower post-intervention activity levels.

Contrary to the hypothesis, there was little change in most secondary outcomes in the yoga group. However, considering the qualitative findings, this may be because changes were idiosyncratic to individuals. A subtheme that emerged was that the participants reported a range of health improvements that were relevant to them, including pain, cough, sleep, fatigue and wellbeing.

There was no statistically or clinically significant change in SGRQ for the control group, yet group median moderate-vigorous physical activity increased from 14 to 44 min/day (average within-person increase of 11 min) and within-person step-count increased by 682 steps/day. Thus, perhaps interventions that increase physical activity alone may be insufficient for improving multifaceted outcomes such as health-related quality of life. Nevertheless, generally physical activity is positively correlated with good mental and physical health [45]. Very few studies characterise physical activity in people with severe asthma objectively. We recently published the first objective evidence that people with severe asthma walk over 2000 fewer steps/day than healthy controls, reaching only 5362 steps/day, and record a median of 22 min/day of moderate-vigorous physical activity [3]. In the current study, the control group recorded 44 min/day post-intervention. Therefore, participants were on average achieving above national guidelines of 150 min of moderate-vigorous activity/week [20]. Achieving national benchmarks for physical activity is therefore feasible for people with severe asthma. Using an activity tracker alongside coaching toward a step goal may be an effective way to increase physical activity in people with severe asthma and should be explored.

In terms of feasibility and acceptability, this was a twice-a-week, closed-group programme of yoga delivered over 16-weeks. It was designed to be suitable regardless of experience and physical limitations, with the exercises supervised and modified as required. Participants appreciated modifications the instructor made and the “gentle”, “achievable” nature of the exercise. The classes did not increase dyspnoea and resulted in few adverse events, highlighting this as a feasible exercise for people with severe asthma. Participants reported that the structure facilitated the experience, allowing them to gradually build skills and providing a sense of commitment that they found motivating. Nevertheless, the structure was limiting for people who worked, were unwell or injured, or had unexpected commitments arise during the programme. Participants completed a median of 19.5/32 sessions. During interviews participants expressed disappointment and frustration at being unable to attend the programme. Among individuals approached for the study, being unable to commit to the intervention was a common reason for not participating. These are important considerations for designing feasible exercise interventions, highlighting a need to balance providing a sense of commitment with a programme flexible enough to accommodate a diverse and sometimes unwell population.

There were two other important considerations relevant to developing and implementing exercise interventions for people with severe asthma. Firstly, participants appreciated the social connection the intervention provided. Social connection and norming are a well-established stimulators of health behaviour change [46]. Secondly, participants appreciated the intersection between multiple components. For example, combining breath, movement and relaxation during the yoga classes, combining an exercise class with an activity tracker for outside class, and combining an exercise intervention with their severe asthma health care. The desirability for and benefits of a “package” of different elements has been described in other qualitative work regarding preferences for medication models of care in severe asthma [47].

There are several limitations to consider when interpreting these findings. Firstly, this was a small pilot trial designed to assess feasibility and acceptability, inform future studies and aid implementation into practice. Low sample size reduced our power to detect statistically significant effects, such that the primary outcome effect was only detected through planned comparisons. A larger sample size would increase power and verify whether the findings are robust. Further a future study should also include longer term follow up in a larger sample to determine the maintenance of effect. Nevertheless this study remains important, as there are few studies that have tested physical activity interventions specifically in a severe asthma population. The strengths of this study are the combination of the quantitative assessments in two active intervention groups, and the rich qualitative data that informs patient acceptability and preferences. These data should be used to inform future severe asthma physical activity RCTs. We acknowledge that behavioural interventions, such as the current intervention, performed unblinded using volunteers are subject to selection and measurement bias. Participants entered the study knowing the intervention was a yoga trial, which may have led to selection bias in the type of participants who volunteered. Furthermore, participants knew their group allocation, which may have led to bias in participant response to outcome questionnaires. To reduce these risks, we ensured both groups were informed that the effect of this intervention is unknown, and that true equipoise exists. The patient information statement was presented objectively to reflect the current evidence. To reduce the chances of contamination between arms, scheduled appointments for the control and intervention groups were on different days.

A yoga and mindfulness intervention is feasible, acceptable and improved health-related quality of life for people with severe asthma. These data are useful in informing larger RCTs to appropriately test the efficacy of the approach. From the patient’s perspective, exercise interventions for people with severe asthma may benefit from incorporating opportunities for social connection, having a flexible programme structure, incorporating a shift in mindset, addressing breathing and asthma symptoms, and having multiple synergistic components. Studies to improve physical activity among people with severe asthma are needed, and this study is important in informing the design of future research.

## Supplementary Information

**Additional file 1**. Supplementary methods and additional results.

## Data Availability

The datasets used and/or analysed during the current study are available from the corresponding author on reasonable request.

## Abbreviations

ACQ: Asthma Control Questionnaire
ATS: American Thoracic Society
COPD: Chronic obstructive pulmonary disease
ERS: European Respiratory Society
FeNO: Fractional exhaled nitric oxide
RCT: Randomised controlled trial
REML: Restricted maximum likelihood
SD: Standard deviation
SGRQ: St George’s Respiratory Questionnaire
