# Supplementary material for: A feasibility randomised controlled trial of Novel Activity Management in severe ASthma-Tailored Exercise (NAMASTE): yoga and mindfulness
Source: BMC Pulm Med. 2021 Feb 27;21:71. doi: 10.1186/s12890-021-01436-3 (PMC7913205; doi:10.1186/s12890-021-01436-3)
Supplement: Supplementary file 1 — Additional file 1. Supplementary methods and additional results. [file 12890_2021_1436_MOESM1_ESM.pdf]

Additional File 1: Supplementary methods and additional results

A feasibility randomised controlled trial of Novel Activity Management in severe ASthma – Tailored Exercise (NAMASTE): Yoga and Mindfulness

Sarah A Hiles[1]

Paola D Urroz[1]

Peter G Gibson[1,2]

Adam Bogdanovs[3]

Vanessa M McDonald[1,2]

Affiliations

[1] Centre of Excellence in Severe Asthma & Priority Research Centre for Healthy Lungs, University of Newcastle, NSW, Australia.

[2] Department of Respiratory and Sleep Medicine, John Hunter Hospital, NSW, Australia.

[3] Yoga For All, Newcastle, NSW, Australia.

Methods: Additional detail

Inclusion criteria

We included adults (≥18 years old) with evidence of variable airflow limitation in the last 10 years and severe asthma according to the European Respiratory Society (ERS)/American Thoracic Society (ATS) taskforce definition:(1) asthma requiring high-dose inhaled corticosteroids (ICS) (>1000µg beclomethasone equivalent(2)) with a second controller to prevent uncontrolled disease or disease that remains uncontrolled despite therapy. Second controller included long-acting beta agonists (LABA); and/or long-acting muscarinic antagonists (LAMA), and/or maintenance oral corticosteroids (OCS) ≥50% of the past year; and/or montelukast; and/or theophylline. Uncontrolled asthma was defined as Asthma Control Questionnaire (ACQ)(3) scores ≥1.5; frequent severe exacerbation (≥2 systemic corticosteroids of 3 or more days in the previous year); serious exacerbation in the previous year (hospitalisation, intensive care unit stay, mechanical ventilation); and/or persistent airflow limitation (pre-bronchodilator forced expiratory volume in 1 second [FEV_1] <80% predicted in the face of a reduced FEV1/forced vital capacity [FVC]). Exclusion criteria were pregnancy; high dependence on medical care; significant life-limiting comorbidity; current lung cancer or other blood, lymphatic or solid organ malignancy; primary diagnosis of a lung disease other than asthma; cognitive impairment, poor English language skills or significant untreated hearing impairment that prevented completion of data collection forms or understanding verbal instructions; inability to attend study visits or intervention classes; current

participation in any other clinical trial; and/or comorbidity that would place the individual at substantial risk of harm or interfere with their ability to participate in the intervention. Participants who had an exacerbation (OCS initiation or increase from maintenance dose for ≥3 days) in the previous four weeks had their baseline visit postponed.

Intervention (yoga and mindfulness) programme

Participants randomised to the intervention group participated in two supervised 75-minute group classes of yoga and mindfulness per week for 16 weeks in a private room at the Hunter Medical Research Institute (up to 8 participants/class), in addition to usual care. The timetable included a 2:05pm and 3:35pm option running on Mondays and Fridays. Participants had the choice to attend one of the two classes that were scheduled on each of the two days. An accredited yoga practitioner designed and taught the sessions. The intervention was designed to be suitable for all participants regardless of experience, ability and physical limitations.

The classes focused on three key concepts: increasing movement, controlling breath, and meditation activities to improve mindfulness. Participants received instruction throughout the class on how to perform the yoga exercises and precautions for participating in each exercise. Equipment was provided to assist the participants during the class. This included yoga mats, bolsters, blankets and chairs. The classes provided the opportunity for participants to learn and practice the skills, with the explicit aim of applying the lessons at home.

During the yoga and mindfulness classes, participants were taught the following practices:

1. Asana (movement - physical practices):

The postures included forward bends, back bends, side bends and spinal twists in standing, sitting or lying positions. Balancing practices were included to develop strength, confidence, stability, focus and a sense of calm. A ‘flow’ of practices was taught individually and then put together in a gentle, moving sequence.

2. Pranayama (breath):

A series of simple breathing practices was taught with the intention of deepening the ‘yogic’ experience rather than any real focus on alleviating asthma related issues. Practices such as abdominal breathing, thoracic breathing, full yogic breath (a combination of abdominal and thoracic), ujjayi breath and alternate nostril breathing to bring awareness, balance, focus and calm to an individuals’ practice.

3. Meditation (focus, concentration, relaxation, stillness, awareness):

The meditation practices are an extension of the posture and breathing practices. These practices included observing the natural breath, counting the breath, the layering of mental anchors to still and focus the mind. A guided, relaxation practice of Yoga Nidra was performed lying comfortably on the floor.

It started as a 10-minute practice, evolving into a full, 30-minute practice of deep relaxation on Fridays.

In addition to attending the scheduled classes, the yoga instructor provided participants with information on how to practice the yoga and mindfulness skills at home.

The safety of participants was considered by having an experienced clinical researcher present at the start and end of the yoga sessions to monitor participant’s ability to participate in the class and was on call throughout the session in case of adverse events. A summary of each participant’s relevant medical history including any musculoskeletal injuries/conditions was made available to the yoga instructor and clinical researcher during the yoga sessions. An emergency trolley equipped with oxygen and disposable nasal prongs, salbutamol with spacer and monitoring equipment for vital signs (blood pressure, oxygen saturation and heart rate) was readily available in the room throughout every class. Participants were advised to self-administer salbutamol prior to commencement to prevent exercise-induced bronchospasm. Participant’s self-ratings on the BORG Dyspnoea Scale were recorded at the start and end of each session.

During the first yoga class, participants received an activity tracker (Fitbit Charge 2 [Fitbit, Inc], or pedometer (Yamax Digi Walker SW700 Pedometer) for participants without smartphones) and were encouraged to increase their daily

steps each day until they reached a goal of 10,000 steps/day. The aim was to increase steps per day, but not necessarily reach the 10,000 step/day goal. The Mendoza algorithm for step progression was provided to participants to guide their step goal.(4) Participants were also given several information sheets regarding the Australian Government physical activity guidelines for adults,(5) SMART goal-setting,(6) exercise and severe asthma,(7) and practicing mindfulness.(8) They were also given a printed calendar as a way to track their activity goals and a copy of the BORG Dyspnoea Scale to use as a tool when monitoring dyspnoea during physical activity.

Control group programme

Participants randomised to the control group received a minimal physical activity goal-setting intervention supported through telephone contact, in addition to usual care. Participants were mailed an activity tracker and informed to record their steps per day during an initial 7-day period using a log provided. Other materials provided included the same printed documents as the yoga group (information sheets, printed calendar and BORG Dyspnoea Scale), and a workbook to use during telephone sessions to record goals and progress toward goals.

During the first call with a clinical research officer (average 14:27 minutes), the participant set SMART goals to achieve within the 16-week period. The first goal was targeting steps per day according to the Mendoza algorithm.(4) To set their steps per day goal a baseline was deemed as their average steps per day

during the initial 7-day period. The baseline average steps per day was then applied to the Mendoza algorithm to calculate a recommended increase of steps per day. Participants were given the option to set a second goal of their choosing relating to physical activity or another aspect of wellbeing.

The research officer scheduled re-occurring telephone coaching sessions every 2 weeks for 16 weeks with the participant (average 7:17 minutes/call). The aims of these telephone coaching sessions were to check on progress toward goals, encourage the participant and readjust goals if necessary.

All participants set a second goal, alongside their step count goal. Three participants revised their goal during the follow-up phone calls. The most commonly set goals related to completing a particular exercise goal (e.g., attending an exercise class; 5/11 goals set) or increasing physical activity in general (e.g., amount of moderate-to-vigorous activity per week; 4/11 goals set).

Results: Additional detail

Participant experience of the yoga intervention

The qualitative analysis revealed five core themes regarding the participant's views on the feasibility and acceptability (barriers and facilitators) of the intervention. We focus on experiences that will aid translation into practice and in the design of future exercise interventions for severe asthma.

Social connection

A connection with others in the class, the instructor and the broader community facilitated participant's positive experiences of the intervention. Firstly, the group setting assisted learning and allowed participants to benchmark their abilities and progress against others at a similarly beginner level with similar health characteristics. That this was a class specifically for beginners of a similar (perceived) age group with severe asthma severity was recommended highly. Some participants described the group setting as fun and reported decreased social isolation. The fact the class was severe asthma-specific was highly valued. Participants enjoyed speaking with others about their illness/medication experiences, they felt less self-conscious about symptoms such as cough, and they believed the shared experience of severe asthma enhanced togetherness and supportiveness. Secondly, all participants expressed that the instructor was essential to their experience. The teacher's attitude, personality, attention, explanations and feedback enhanced learning and facilitated a positive class environment, increased retention and instilled a desire to do home practice. However, participants expressed hesitation at attending a class with a different

instructor, which may be a barrier to continued practice. Finally, the intervention seemed to facilitate a connection with the broader community. Participants were able to talk about their experiences with yoga, or even practice yoga, with friends, family and others, in a way that may not have been possible with an exercise less well-known in the community.

Setting and commitment

The structured, closed-group programme generated facilitators and barriers. The participants appreciated that the programme gradually built on skills such that they could see changes in their ability over time, which enhanced their confidence. They felt a commitment to attend, or that it became a habit/routine to attend, which facilitated retention. However, several participants described disappointment and frustration at not being able to attend due to illness or other commitments, which was a barrier to continued practice. Some wanted a longer programme to make up for missed attendance. Participants suggested that working may be a barrier to attendance; participants who worked had to fit the class around their schedules, which they sometimes found logistically challenging or caused them to feel “rushed”. Another important factor for participants was that this was a research study. They prioritised attendance because it was a research study; they wanted to contribute to knowledge, trusted the people who put the programme together, and appreciated that it was conducted at a “safe” setting (i.e., a medical research centre during office hours). Although the context of a research study increased initiation and

retention, for some it was a barrier to ongoing practice as they viewed participating as a “one-time-thing”.

Changed mindset

Participants commonly expressed increased “confidence”, “motivation” and a more positive attitude. Participants reported confidence to try exercises or activities of daily living that they usually would not attempt, or push themselves further in their activity than they usually would (e.g., initiating or increasing distance of walking). They also reported increased motivation or “will” to exercise or do daily activities, often ascribed to increased awareness of their physical activity levels (e.g., via the Fitbit or simply feeling “more active”) or awareness of the positive effects they experienced from physical activity. These experiences were particularly common among people who recently started on monoclonal antibody therapy, which had improved their asthma control. Regarding yoga specifically, participants described new-found confidence to undertake yoga at home or in a community setting, and all participants reported incorporating at least one aspect of the yoga in their daily routine. Many had pre-conceived ideas about yoga; the yoga exceeded their expectations and shifted attitudes - they developed an understanding of yoga and saw why it might be beneficial for asthma. Many participants spoke about having a more positive outlook on life and decreased worry. The class and instructor in particular helped people achieve a sense of “calm”. For some, they felt reduced awareness of their physical sensations during the class (particularly relaxation)

whereas others reported heightened awareness and focus, usually after class had finished.

Addressing breathing and asthma symptoms

One of the most commonly perceived benefits was “improved breathing”. This was ascribed to multiple components of the intervention. Firstly, many found the focus on connecting breath with movement particularly helpful for managing day-to-day activity and intentional exercise. In particular, they thought that the improved posture was helpful to broadening the chest and improving breathing. Participants liked that there were able to “do” this exercise and found it gentle, since the content of the class was modified to suit the needs of the participants. Secondly, some found the focus on relaxation/stress reduction helpful for the “emotional side” of severe asthma and reducing anxiety around symptoms of breathlessness, cough and wheeze. Participants believed that the relaxation techniques would be particularly useful during an acute asthma attack, and reported increased confidence and sense of control about managing asthma symptoms. Thirdly, some in the class connected the learning in the yoga class with previous experience of breathing exercises (e.g., speech therapy, breath retraining), which they used as a tool to achieve more physical activity.

Intersection of different elements

There were five main facilitators here. Firstly, participants felt the combination of breath, relaxation and exercise/movement beneficial. Secondly, they found the combination of the Fitbit and yoga beneficial; where the Fitbit brought

awareness of physical activity or motivated people to start their own new physical activity outside, and yoga was a way to learn different skills that facilitated physical activity outside of class. Thirdly, the intervention alongside their medication (either a recent or concurrent change in medication) was helpful; they used the intervention as a way to get back to being physical activity now that they felt more well than they had in a long time. Participants reflected that if they felt unwell, they were uncertain whether they could keep up with the classes or exercises. Finally, that the programme addressed not just their severe asthma, but also had benefits for a range of comorbidities. This experience of improvements in comorbidity was highly personal. Commonly identified effects that weren't assessed in our quantitative data were improved "strength", "flexibility", "posture", "endurance", "vigor" and improved sleep through the application of relaxation techniques. People reported improvements in comorbidity, but also that aspects of comorbidity impeded their ability to follow the class. Pain was the biggest barrier to participating and ongoing practice. The tailored approach allowed them to overcome physical barriers; participants particularly appreciated the instructor being responsive to the needs of the class.

References

1. Chung KF, Wenzel SE, Brozek JL, Bush A, Castro M, Sterk PJ, et al. International ERS/ATS guidelines on definition, evaluation and treatment of severe asthma. European Respiratory Journal. 2014;43(2):343-73.

2. Global Initiative for Asthma. Global Strategy for Asthma Management and Prevention Fontana, USA: Global Initiative for Asthma; 2019 [cited November 24 2019]. Available from: https://ginasthma.org/gina-reports/.

3. Juniper EF, Svensson K, Mörk AC, Ståhl E. Measurement properties and interpretation of three shortened versions of the asthma control questionnaire. Respiratory Medicine. 2005;99(5):553-8.

4. Mendoza L, Horta P, Espinoza J, Aguilera M, Balmaceda N, Castro A, et al. Pedometers to enhance physical activity in COPD: a randomised controlled trial. The European respiratory journal. 2015;45(2):347-54.

5. Australian Government Department of Health. Australia's Physical Activity and Sedentary Behaviour Guidelines for Adults Canberra, Australia: Commonwealth of Australia; 2014 [cited 2019 January 14]. Available from: https://www1.health.gov.au/internet/main/publishing.nsf/Content/health-pubhlth-strateg-phys-act-guidelines#npa1864.

6. LiveLighter. Factsheet - Goal Setting Subiaco, Australia: LiveLighter; 2018 [cited 2019 January 14]. Available from: https://livelighter.com.au/Tools-and-Resources/Resources.

7. Asthma UK. Exercising when you have severe asthma London, UK: Asthma UK; 2016 [cited 2019 January 14]. Available from: www.asthma.org.uk/advice/severe-asthma/making-life-easier-severe-asthma/exercising-when-you-have-severe-asthma/.

8. ReachOut Australia. How to practise mindfulness Pyrmont, Australia: ReachOut Australia; 2018 [cited 2019 January 14]. Available from: http://au.reachout.com/articles/how-to-practise-mindfulness.
